# Patients with Blepharitis Are at Elevated Risk of Anxiety and Depression

**DOI:** 10.1371/journal.pone.0083335

**Published:** 2013-12-30

**Authors:** Chun-Chi Chiang, Cheng-Li Lin, Yi-Yu Tsai, Chiao-Ling Peng, Ya-Tang Liao, Fung-Chang Sung

**Affiliations:** 1 Department of Ophthalmology, China Medical University Hospital, Taichung, Taiwan; 2 School of Medicine, China Medical University, Taichung, Taiwan; 3 Management Office for Health Data, China Medical University Hospital, Taichung, Taiwan; 4 Department of Public Health, China Medical University College of Public Health, Taichung, Taiwan; Federal University of Rio de Janeiro, Brazil

## Abstract

**Purpose:**

Population-based cohort study on the risk of anxiety and depression in patients with blepharitis is limited. This study evaluated whether blepharitis patients are at a higher risk of anxiety and depression.

**Design:**

A retrospective cohort study.

**Methods:**

We used the universal insurance claims data from 1997 to 2010 in Taiwan to identify annually patients with newly diagnosed blepharitis (N = 9764) and without the disease (N = 39056). Incidences, rate ratios (IRR) and hazard ratios (HR) of anxiety and depression were measured for both cohorts by baseline demographic characteristics and comorbidities until the end of 2010.

**Results:**

Compared with the non-blepharitis cohort, the blepharitis cohort had higher incidence of anxiety (15.9 vs. 9.5 per 1000 person-years), with an adjusted HR of 1.58 (95% confidence interval (CI) = 1.46–1.70). The incidence of depression was also higher in the blepharitis cohort (7.66 vs. 5.05 per 1000 person-years), with an adjusted HR of 1.42 (95% CI = 1.28–1.58). The blepharitis cohort to the non-blepharitis cohort IRR decreased from 1.73 in the first quartile to 1.32 in the 4^th^ quartile for anxiety, and from 1.67 to 1.29 for depression.

**Conclusions:**

Patients with blepharitis are at elevated risks of anxiety and depression. The risk is higher in earlier period after the diagnosis of blepharitis, and declines by time, but remains significantly higher for those with blepharitis than those without blepharitis.

## Introduction

Characterized by inflamed eyelids, blepharitis is a high prevalent ophthalmic complains difficult to cure in eye care practices[1]. Crust or flakes on eyelashes, stuck eyelids, reddish eyelids, and blurred vision are the common signs and symptoms[2]. The onset of blepharitis can be acute, other than the more generally form of long standing chronic inflammation. Staphylococcal infection, seborrheic, and meibomian gland dysfunction (MGD) are major etiological cause of these conditions[3]. Patients suffer discomfort evaporative dry eye disease are mainly associated with MSG because of ocular surface inflammation[2].The ocular surface condition may further exacerbate due to immune-mediated inflammatory processes[4]. The persistent discomfort eyes, and unattractive appearance, and uneasy feeling may precipitate psychological stress and negative social implications for patients, including depressed and anxious mood[1].

The WHO World Health Survey shows that patients with chronic diseases are more likely to have comorbidity of depression, particularly in patients with angina, arthritis, asthma, and diabetes[5]. For patients with blepharitis, in addition to the burden of the disease and vision disturbance, there are other factors that may contribute to the risk of psychologic condition. The drugs used to treat inflammatory diseases, such as corticosteroids, have been associated with the risk of mania, depression, and other behavioral changes[6]. There are also evidences that cytokines are able to cross the blood-brain barrier linking with behavioral changes in patients with chronic inflammation[7], [8]. A combination of these factors may place patients with ocular inflammatory diseases at an elevated risk for mood dysfunction.

Because of uncomfortable and distressing conditions, we hypothesized that patients with blepharitis would be at an increased risk of mental illness including major depression and anxiety. No large study has ever evaluated the association using prospective design. This study conducted a retrospective follow-up observation to estimate the risk of depression and anxiety in patients with blepharitis using a nationwide insurance dataset in Taiwan.

## Methods and Materials

### Study Design and Data Source

We obtained a representative claims data of one million insured people in the National Health Insurance Research Database (NHIRD). This universal health insurance program has covered nearly 99% of the 23.74 million residents and contracted with 97% of the hospitals and clinics for comprehensive health care in Taiwan[9]. In this study, patients with blepharitis were identified from the NHIRD inpatient and outpatient expenditure claims data files from 1997 until the end of 2010. These files contained the information on demographic status (birth date and sex) of insured population and services provided, and reimbursements for the services. The diagnoses of diseases used the International Classification of Disease, 9th Revision, Clinical Modification (ICD-9-CM). For the confidentiality, patient identifications were scrambled according to the Department of Health regulations to strengthen data security and protect patient's privacy. This study was exempted from full ethical review (IRB permit number: CMU-REC-101-012).

### Study Subjects And Comorbidities

Patients who had made at least three outpatient visits with diagnoses of blepharitis (ICD-9-CM code 373.0) were eligible for the study and identified annually from 1997 to 2010 for the blepharitis cohort. The first diagnosis date was defined as index date for estimating the follow-up time. For each blepharitis patients, 4 persons free from the disease were identified from the rest of claims data for the non-blepharitis cohort, with age, sex and index year frequency matched. Patients with the history of depression (ICD-9-CM: 300.00, 296.2, 296.3, 300.4 and 311) or anxiety (ICD-9-CM: 300.00) before the index date or with missing information on age or sex were excluded. The diseases considered to be comorbidities included hypertension (ICD-9- CM codes 401 to 405, A260, A269), diabetes (ICD-9-CM codes 250, A181), hyperlipidemia (ICD-9-CM codes 272, A182), coronary artery disease (ICD-9-CM codes 410 to 414, A270, A279) and stroke (ICD-9-CM codes 430 to 438, A290 to A299).

### Statistical Analysis

Chi-square test was used to examine the distributions of baseline demographic characteristics and comorbidities between blepharitis and non-blepharitis cohorts. Follow-up person-years were estimated for all subjects in both cohorts until depression or anxiety identified, or censored because of loss to follow-up or withdrawn from the insurance, or the end of 2010. Incidence rates of depression and anxiety were estimated by demographic status and comorbidities. The blepharitis cohort to the non-blepharitis cohort incidence rate ratio (IRR) and 95% confidence interval (CI) were calculated using Poisson regression analysis. Cox proportional hazards regression analysis was also used to measure the hazard ratios (HR) and 95% confidence intervals (CI) of depression and anxiety associated with blepharitis, adjusting for covariates. We also depicted the blepharitis cohort to the non-blepharitis cohort IRR of depression and anxiety by follow-up years in quartiles. A *p* value of less than 0.05 was considered to be statistically significant. All analyses were performed using the SAS statistical package for Windows, version 9.2 (SAS institute, Inc., Cary, NC, USA).

## Results

This study identified 9764 persons for the blepharitis cohort and 39056 persons for the non-blepharitis cohort. Both cohorts were similar in age and sex distributions with a mean age of 54.9±18 years and near 60% of subjects were women (Table 1). Compared with the non-blepharitis cohort, patients in the blepharitis cohort were more prevalent with hypertension, diabetes mellitus, hyperlipidemia, stroke and coronary artery disease.

**Table 1:** Demographic characteristics and comorbidities in blepharitis cohort and non-blepharitis cohort.

	Blepharitis		
	No	Yes	
Variable	N = 39056	N = 9764	*p*-value&
Sex	n(%)	n(%)	
Female	23756(60.8)	53939(60.8)	0.99
Male	15300(39.2)	3825(39.2)	
Age, years			
Mean±SD	54.9±18.0	54.8±18.0	0.68
20–39	9320(23.9)	2331(23.9)	0.70
40–64	15009(38.4)	3711(38.0)	
65+	14727(37.7)	3722(38.1)	
Comorbidity			
Diabetes	5512(14.1)	1721(17.6)	<0.0001
Hypertension	12936(33.1)	3658(37.5)	<0.0001
Hyperlipidemia	6892(17.7)	2349(24.1)	<0.0001
Coronary artery disease	6165(15.8)	2028(20.8)	<0.0001
Stroke	4251(10.9)	1288(13.2)	<0.0001

&:Chi-square test; t-test used to test means.

The incidence rate of anxiety was 1.67-fold higher in the blepharitis cohort than in the non-blepharitis cohort (15.9 vs. 9.5 per 1000 person-years), with an adjusted HRs of 1.58 (95% CI = 1.46–1.70) (Table 2). So was the incidence of depression 1.52-fold higher in the blepharitis cohort (7.66 vs. 5.05 per 1000 person-years), with an adjusted HRs of 1.42 (95% CI = 1.28–1.58). The incidence rates of both anxiety and depression were higher in women than in men, and increased with age in both cohorts. The gender and age differences in the incidence rates of anxiety were greater than that of depression. However, the age-specific blepharitis cohort to non-blepharitis cohort IRR of anxiety were higher in those in less 40 years of age, with an adjusted HR of 1.97 (95% CI = 1.59–2.43). The adjusted HR reduced to 1.56 (95% CI 1.39, 1.75) for the elderly.

**Table 2:** Incidence rates by sex, age and cohort, and blepharitis cohort to non-blepharitis cohort rate ratios and Cox model measured hazard ratios.

	Blepharitis							
	No			Yes				
Variables	Event	PY	Rate^#^	Event	PY	Rate^#^	IRR*(95% CI)	Adjusted HR^†^ (95% CI)
Anxiety	2208	232509	9.50	932	58652	15.9	1.67(1.59, 1.76)**	1.58(1.46, 1.70)**
Sex								
Female	1575	143637	11.0	638	35934	17.7	1.62(1.51, 1.73)**	1.53(1.40, 1.68)**
Male	633	88871	7.12	294	22718	12.9	1.82(1.67, 1.98)**	1.68(1.46, 1.93)**
Stratify age								
20–39	262	59831	4.38	131	15062	8.70	1.99(1.78, 2.22)**	1.97(1.59, 2.43)**
40–64	1003	95058	10.5	394	23232	17.0	1.61(1.48, 1.75)**	1.50(1.33, 1.68)**
65+	943	77620	12.1	407	20358	20.0	1.65(1.51, 1.79)**	1.56(1.39, 1.75)**
Depression	1199	237529	5.05	468	61066	7.66	1.52(1.43, 1.61)**	1.42(1.28, 1.58)**
Sex								
Female	832	147335	5.65	297	37696	7.88	1.43(1.33, 1.53)**	1.32(1.16, 1.51)**
Male	367	90194	4.07	171	23371	7.32	1.82(1.68, 1.98)**	1.63(1.36, 1.96)**
Stratify age								
20–39	214	59993	3.57	79	15318	5.16	1.47(1.31, 1.64)**	1.44(1.11, 1.87)**
40–64	500	97717	5.12	180	24318	7.40	1.47(1.35, 1.61)**	1.36(1.15, 1.62)**
65+	485	79818	6.08	209	21430	9.75	1.64(1.51, 1.78)**	1.48(1.26, 1.74)**

PY, person-years; Rate^#^, incidence per 1,000 person-years; IRR*, incidence rate ratio.

Adjusted HR^†^: adjusted for age, sex, diabetes, hyperlipidemia, hypertension, coronary artery disease and stroke.

*p<0.05.

**p<0.01.

**Figure 1:**
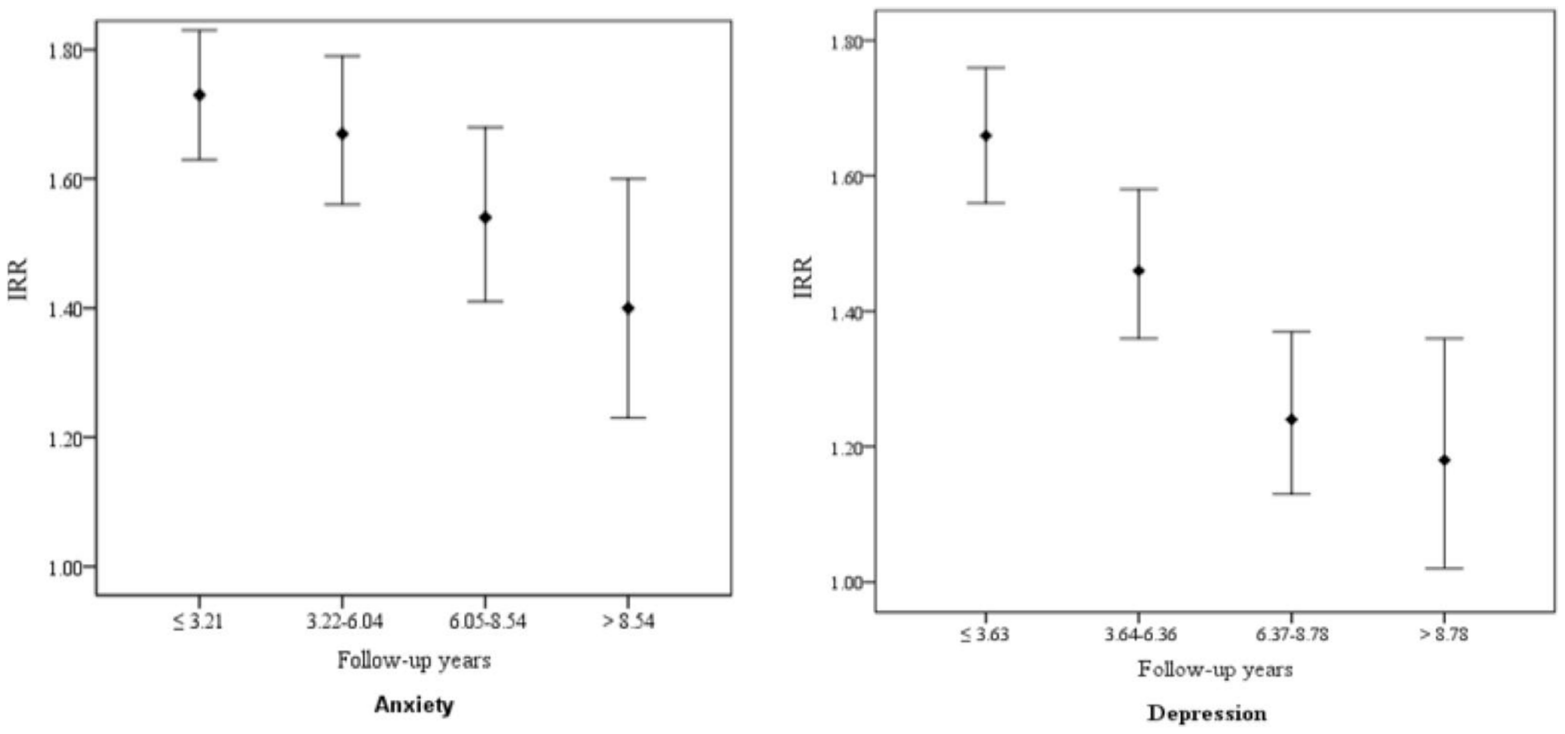
Blepharitis cohort to non-blepharitis cohort incidence rate ratio of anxiety and depression by quartile of follow-up years.

The comorbidity specific analysis in the multivariable Cox proportional hazards regression model showed consistently higher incidence rates of anxiety and depression in the blepharitis cohort than in the non-blepharitis cohort (Table 3). The incidence increased further in blepharitis patients with comorbidity, particularly in those had a stroke. Figure 1 shows that the blepharitis cohort to the non-blepharitis cohort IRR for both anxiety and depression declined by follow-up time, from 1.73 in the first quartile to 1.40 in the 4^th^ quartile for anxiety, and from 1.67 to 1.18 for depression.

**Table 3:** Incidence of depression and anxiety, blepharitis cohort to non-blepharitis cohort rate ratio and Cox model measured hazards ratio by comorbidity.

	Blepharitis							
	No			Yes				
	Event	PY	Rate^†^	Event	PY	Rate^†^	IRR*(95% CI)	Adjusted HR^†^ (95% CI)
Anxiety								
Comorbidity								
No	857	141785	6.04	332	31190	10.64	1.76(1.63, 1.90)**	1.76(1.55, 1.99)**
Yes	1351	90724	14.89	600	27462	21.85	1.47(1.36, 1.58)**	1.45(1.32, 1.60)**
Diabetes								
No	1832	204332	8.97	746	49355	15.11	1.69(1.59, 1.79)**	1.59(1.46, 1.73)**
Yes	376	28176	13.34	186	9297	20.01	1.50(1.32, 1.71)**	1.48(1.24, 1.76)**
Hypertension								
No	1153	163805	7.04	488	38450	12.69	1.80(1.69, 1.93)**	1.70(1.53, 1.89)**
Yes	1055	68703	15.36	444	20203	21.98	1.43(1.31, 1.56)**	1.42(1.27, 1.58)**
Hyperlipidemia								
No	1624	196174	8.28	636	45767	13.90	1.68(1.58, 1.78)**	1.63(1.49, 1.79)**
Yes	584	36335	16.07	296	12886	22.97	1.43(1.28, 1.60)**	1.44(1.26, 1.66)**
Coronary artery disease								
No	1624	201095	8.08	657	47786	13.75	1.70(1.61, 1.81)**	1.66(1.52, 1.82)**
Yes	584	31413	18.59	275	10867	25.31	1.36(1.21, 1.53)**	1.37(1.18, 1.58)**
Stroke								
No	1861	212124	8.77	752	51842	14.51	1.65(1.56, 1.75)**	1.58(1.45, 1.72)**
Yes	347	20385	17.02	180	6810	26.43	1.55(1.34, 1.79)**	1.54(1.29, 1.85)**
Depression								
Comorbidity								
No	523	143223	3.65	170	31998	5.31	1.45(1.34, 1.58)**	1.45(1.22, 1.72)**
Yes	676	94306	7.17	298	29069	10.25	1.43(1.32, 1.55)**	1.39(1.22, 1.60)**
Diabetes								
No	992	208513	4.76	364	51376	7.09	1.49(1.40, 1.59)**	1.41(1.25, 1.59)**
Yes	207	29016	7.13	104	9691	10.73	1.50(1.31, 1.73)**	1.46(1.15, 1.85)**
Hypertension								
No	668	166162	4.02	241	39710	6.07	1.51(1.40, 1.63)**	1.45(1.25, 1.68)**
Yes	531	71367	7.44	227	21356	10.63	1.43(1.30, 1.57)**	1.38(1.18, 1.62)**
Hyperlipidemia								
No	915	199653	4.58	319	47360	6.74	1.47(1.38, 1.57)**	1.41(1.24, 1.60)**
Yes	284	37876	7.50	149	13706	10.87	1.45(1.28, 1.64)**	1.45(1.19, 1.77)**
Coronary artery disease								
No	894	204529	4.37	326	49435	6.59	1.51(1.41, 1.61)**	1.47(1.29, 1.67)**
Yes	305	33000	9.24	142	11632	12.21	1.32(1.16, 1.50)**	1.31(1.07, 1.60)**
Stroke								
No	993	216354	4.59	349	53930	6.47	1.41(1.32, 1.50)**	1.37(1.20, 1.53)**
Yes	206	21175	9.73	119	7136	16.68	1.71(1.48, 1.99)**	1.68(1.34, 2.11)**

Rate^#^, incidence rate, per 1,000 person-years; IRR*, incidence rate ratio.

Adjusted HR^†^: adjusted for age, sex, diabetes, hyperlipidemia, hypertension, coronary artery disease and stroke.

*p<0.05.

**p<0.01.

## Discussion

Blepharitis is a common ocular disease that has been associated with many systemic medical conditions. In an age and gender matched retrospective case-control study (n = 16706 each group), Nemet et al found that patients with blepharitis are prevalent with not only psychologic conditions, but also systemic conditions and other eye conditons[10]. A survey conducted in the US showed that approximately 37% of patients seen by ophthalmologists and 47% of patients seen by optometrists have complains of blepharitis[2]. Both the ophthalmologic disorder and the associated mood dysfunction are important factors affecting the quality of life of patients. In this study, we found that patients with blepharitis had a 67% higher risk of anxiety and 52% higher risk of depression in the 9-year follow-up period. The risk of anxiety shown in our cohort analysis is consistent with the odds ratio of 1.6 (95% CI 1.4–1.9) found by Nemet et al.[10].

The occurrence of blepharitis and subsequent psychologic conditions have been associated with psychoneuroimmunology and inflammation[7], [8], [11]–[17]. Patients with blepharitis are at higher risk to have other medical conditions, which may also associate the psychologic consequence[10]. Recent studies have shown that chronic inflammatory and cytokines dysregulation are significant factors relate to the pathophysiology of depression and anxiety[18], [19]. Levels of circulating cytokines interleukin (IL)-1beta, IL-6, interferon gamma, and TNF-alpha are higher in depressed patients than in controls[20]. Patients with cardinal inflammation accompany with depression have elevated inflammatory cytokines and their soluble receptors presenting in peripheral blood and cerebrospinal fluid[21], [22]. Concentrations of acute phase proteins, chemokines, adhesion molecules, and inflammatory prostaglandins are also increased in peripheral blood. Eyelids are rich with blood supply. A murine model has also demonstrated that the concentration of inflammatory cytokines in tear is elevated in response to evaporative dry eye stress, which usually results from blepharitis[23]. Therefore, both psychologic disorder and blepharitis may be affected by the inflammatory reactions.

Previous studies have found that women are up to 3 times more likely to experience depressive disorders than men, including depression, anxiety, and co-existing depression and anxiety[24]–[30]. Our results also revealed that the incidence rates of both anxiety and depression were higher in women than in men, which is consistent with previous reports. However, the sex-specific blepharitis cohort to non-blepharitis cohort IRR shows that relative risks of both anxiety and depression are higher for men than for women. We hypothesize the sex hormone may play a role in the gender difference. Increasing evidences have shown that gene expression of the meibomiam glands, dry eye syndrome, MGD, and evaporative dry eyes are associated with sex steroids[31]–[39]. The incidence of anxiety increased markedly with age in our study. The hormonal therapy in postmenopausal women may have an important role leading to blepharitis and mood dysfunction[32], [34], [35], [38].

Previous clinical and community-based studies have suggested that the prevalence of depression, anxiety, and comorbid disorder decline with age[40]–[42]. Different study designs may attribute to the discrepancy between our finding and results from other studies. There are sufficient medical resources and facilities with convenient access to care for insured people in Taiwan. The elderly patients are easily to get the medical attention. This may explain why the incidence rates of depression and anxiety are higher in older people in this study. Compare with non-blepharitis patients, patients with blepharitis suffer from chronic ocular discomfortable and blurred vision, which affect the quality of life, especially for older people. Hence, these patients have higher tendency to develop depression and anxiety. Nevertheless, the age-specific relative risk of anxiety was some what higher for those aged 20–39 years than the elderly in the blepharitis cohort (IRRs: 1.99 vs. 1.65). The young group is the main working force; the blepharitis symptoms such as ocular discomfortable and physical appearance may affect their work performances and social activities, which induce higher anxiety than their peers without blepharitis.

In addition, compared with non-blepharitis cohort, patients in the blepharitis cohort were also more prevalent with hypertension, diabetes mellitus, hyperlipidemia, stroke and coronary artery disease. Studies have demonstrated a clear relationship between inflammation and the development of cardiovascular disease, diabetes, and cancer[43], [44]. Nemet et al also found some major cardiovascular conditions (carotid artery disease, hyperlipidemia, and hypertension) to be associated with blepharitis[10]. Our results are also consistent with their report.

There are few limitations in our study. First, we used International Classification of Diseases codes to identified diagnoses of blepharitis, depression, anxiety and other medical conditions. People with mild symptoms may not go to the doctor resulting undiagnosed cases. Patients with blepharitis might have more clinic visits than those without blepharitis. We conducted a further data analysis and found that the average number of clinic visit was higher in blepharitis patients than in the comparisons [32.3 (SD 24.4) vs. 20.0 (SD 18.1)]. Therefore, blepharitis patients are more likely to be diagnosed and to get treatment for depression and anxiety. Second, although there may be a small number of patients with undetected blepharitis would be categorized as non-blepharitis and would have a small chance of being selected as part of the comparison cohort. The sample size of each cohort was large enough to compromise the bias. However, the results need to be interpreted with caution.

Blepharitis is characterized by discomfort eyes and unattractive appearance, which may cause uneasy feeling and negative social implications for the patients. The present study shows patients with blepharitis are at higher risk of anxiety or depression. Our findings are of clinical importance given the high prevalence of blepharitis in the general population, and the fact that depression and anxiety are disorders readily treatable and often under diagnosed because its symptoms are frequently overlooked.
